# Islet Transplantation Attenuating Testicular Injury in Type 1 Diabetic Rats Is Associated with Suppression of Oxidative Stress and Inflammation via Nrf-2/HO-1 and NF-*κ*B Pathways

**DOI:** 10.1155/2019/8712492

**Published:** 2019-09-05

**Authors:** Xiandong Zhu, Feixia Guo, Hengjie Tang, Chongchu Huang, Gangyin Xie, Tingting Huang, Yonglin Li, Chengyang Liu, Hongwei Wang, Bicheng Chen

**Affiliations:** ^1^Key Laboratory of Diagnosis and Treatment of Severe Hepato-Pancreatic Diseases of Zhejiang Province, The First Affiliated Hospital of Wenzhou Medical University, Wenzhou, 325000 Zhejiang Province, China; ^2^School of Laboratory Medicine and Life Sciences, Wenzhou Medical University, Wenzhou, 325000, China; ^3^Department of Surgery, School of Medicine, University of Pennsylvania, Philadelphia, PA 19104, USA

## Abstract

Testicular structural and functional impairment is a serious complication in male diabetes mellitus (DM) patients that leads to impaired fertility in adulthood. In contrast to other endocrine therapies, islet transplantation (IT) can effectively prevent and even reverse diabetic nephropathy and myocardial damage. However, whether IT can alleviate diabetes-induced testicular injury remains unclear. In this study, we sought to investigate the effect of IT on diabetes-induced testicular damage. A diabetic rat model was established by streptozotocin injection. DM, IT, and insulin treatment (INS) groups were compared after 4 weeks of respective treatment. We confirmed that IT could effectively attenuate diabetes-induced testicular damage and recover sperm counts more extensively compared with INS in diabetic rats. In addition, significantly higher levels of superoxide dismutase (SOD) activity and lower contents of malondialdehyde (MDA) were detected in the testes of the IT group versus diabetic rats. Mechanism studies revealed that IT significantly activates the expression of Nrf-2, HO-1, and NQO-1 and inhibits upregulation of the NF-*κ*B expression in response to DM, while INS only exhibit slight impact on the protein expression. Therefore, we speculate that IT may prevent the progression of testicular damage by downregulating oxidative stress and inhibiting inflammation via Nrf-2/HO-1 and NF-*κ*B pathways.

## 1. Introduction

Diabetes mellitus (DM) is a chronic metabolic syndrome characterized by prolonged hyperglycemia [1] caused by either insulin secretion disorders, a lack of sensitivity of the cells to insulin, or both [2]. Several reports show that long-term hyperglycemia can lead to a series of serious complications [3]. Moreover, diabetes can exert adverse effects on the male genital system with complications including erectile dysfunction, male infertility, abnormal spermatogenesis, and oligospermia [4, 5]. The occurrence and development of testicular injury can seriously affect the quality of life of male diabetic patients [6]. Therefore, the development of appropriate strategies to prevent loss of testicular germ cells and restore integrity of testicular tissue structures could present an indispensable approach to preserve or improve the fertility of young or adult men patients.

The pathogenesis of DM typically involves multiple mechanisms, among which oxidative stress and inflammation are known to play an important role in testicular injury [7]. Although low amounts of free radicals are essential for spermatogenesis and sperm maturation, the testicles are highly susceptible to increases in these species compared with other tissues. Studies have demonstrated that mammalian sperm cells contain high levels of lipids and unsaturated fatty acids [8]. In the diabetic state, protein glycosylation and glucose autooxidation can cause lipid peroxidation (LPO), which further leads to formation of free radicals. Free radicals occurring in this state may play an important role in DNA damage, glycosylation, protein modification reactions, and lipid oxidative modification [9], which, in turn, result in sperm abnormalities. Overexpression of inflammatory cytokines can significantly suppress testosterone synthesis, alter meiotic DNA synthesis in most spermatocytes, and affect spermatogonial differentiation [10, 11]; it can also lead to gonadal dysfunction, particularly the steroidogenic potential of Leydig cells [12].

At present, the main treatment methods of DM, such as insulin or drug therapy (metformin), can improve male reproductive functions; however, they are also with limitations and associated with a variety of side effects [13, 14]. Hence, finding an effective therapy to treat testicular injury with rare adverse reactions remains a challenge.

In recent years, with the improvement of islet isolation methods, islet transplantation (IT) has become an attractive therapeutic alternative for treating diabetic patients with diabetic complications [15, 16]. Recent studies have shown that IT can regulate the expression of insulin in vivo, stabilize blood glucose regulation, delay progression of clinical symptoms of diabetes, and even reverse related renal and myocardial damage [17–20]. However, the effects of IT on the genital system of diabetic male patients remain unelucidated.

The present study is aimed at investigating the effect of IT on testicular function and elucidating potential molecular mechanisms of its influence on testicular damage using a diabetes-induced rat model. We hypothesize that IT effects this change by modulating antioxidant and anti-inflammatory molecule production via activating the Nrf-2/HO-1 pathway and inhibiting activation of NF-*κ*B.

## 2. Materials and Methods

### 2.1. Animals

A total of 50 healthy mature male Wistar rats (200–220 g) were obtained from the Laboratory Animal Center of Wenzhou Medical University (Wenzhou, China). All the animals were housed in standard environmental conditions and fed water and standard laboratory food for 1 week before their use in the study. All strategies (including the mouse euthanasia procedure) performed on the rats were approved by the institutional animal committee of Wenzhou Medical University for medical laboratory animal sciences.

### 2.2. Animal Model and Study Groups

Diabetic rat models were induced by a single intraperitoneal injection of 50 mg/kg streptozotocin (STZ; Sigma, St. Louis, MO, USA) after an overnight fast. The experimental diabetic rats were confirmed 7 days after STZ injection by measuring blood glucose levels in the blood of the tail vein. Rats with nonfasted blood glucose concentration over 16.67 mmol/l for 3 consequent days were identified as successfully established in this study.

The rats were given twelve weeks to achieve stable diabetic status. They were then randomly divided into four groups: the control group (*n* = 8; nondiabetic), the DM group (*n* = 8; rats were untreated), the INS group (*n* = 8; rats were given insulin (Wanbang Biopharmaceuticals, Jiangsu, China) at a dose of 3 U per injection at 9 a.m. and 9 p.m. every day for 4 weeks), and the IT group (*n* = 8; rats were subjected to IT under the left kidney capsule). All animals were sacrificed 4 weeks after the different treatments, and the left kidney and testicular tissues were collected.

### 2.3. Islet Transplantation

The donor nondiabetic rats were anesthetized with chloral hydrate. The pancreas was carefully separated, and 8 ml of collagenase V was slowly injected into the common bile duct from the opposite direction. Afterward, the pancreas was harvested and digested at 37°C. The islets were purified, centrifuged, and transferred to glass Petri dishes for manual screening. The final purified islets were cultured in Roswell Park Memorial Institute-1640 (Gibco, Carlsbad, CA, USA) at 37°C and 5% CO_2_ for 6 h. Fluorescein diacetate-propidium iodide (FDA-PI) staining was used to assess the viability of purified islets under an inverted fluorescence microscope. The left kidney of the recipient rats was exposed, and 1,000–1,200 islet equivalents (IEQ) of purified islets was slowly transferred under the kidney capsule [21]. Finally, the incision was sutured in layers. Insulin secretion function was evaluated by immunochemistry at 4 weeks posttransplantation.

### 2.4. Histopathology

Left testicular and renal tissues were fixed in 4% formalin, dehydrated in ethanol and xylene, then embedded in paraffin, and sliced to 4 *μ*m thickness sections. After deparaffinization and rehydration, the slides were treated with hematoxylin-eosin (HE) staining to study the testicular and renal pathological changes under a light microscope and analyzed by Image-Pro Plus 6.0 image analysis software. More than 10 fields were evaluated from each section in each group.

### 2.5. Immunohistochemical Examination

Paraffin-embedded testicular and renal tissue sections (thickness: 4 *μ*m) were used for immunohistochemical analysis. To inhibit endogenous peroxidase activity, the sections were incubated with 3% hydrogen peroxide. They were then preincubated in a boiling sodium citrate buffer for antigen retrieval and blocked with 5% normal goat serum for 30 min. Then, the sections were blocked with 5% normal goat serum for 30 min. Primary antibodies for heme oxygenase-1 (HO-1; Abcam, 1 : 200) (Abcam, Cambridge, MA, USA), nuclear factor kappa B (NF-*κ*B; CST, 1 : 200) (Cell Signaling Technology Inc., Danvers, MA, USA), and insulin (CST, 1 : 200) were added to the samples, which were then incubated overnight at 4°C. After washing, the slides were incubated for 1 h with secondary antibodies (goat anti-rabbit antibody) (BioSharp Inc., China). After diaminobenzidine (DAB, brown color, ZSGB-BIO, Beijing, China) and hematoxylin staining under a microscope, the positive regions of sections were analyzed with Image-Pro Plus software. More than 10 fields were evaluated from each section in each group.

### 2.6. Determination of Oxidative Stress Markers

A portion of the right testis was frozen at −80°C. To prepare testicular tissue homogenates for biochemical analysis, the testes were weighed and homogenized, and a homogenate was prepared in cold lysis buffer. MDA levels were evaluated by a Lipid Peroxidation MDA assay kit (Beyotime Biotechnology, China). The principle of this method was to determine the color reaction of thiobarbituric acid (TBA) with MDA by spectrophotometric measurement. The antioxidant enzyme SOD activity was measured depending on a Total Superoxide Dismutase Assay Kit (Beyotime Biotechnology, China). The principle of the method was to inhibit the reduction of nitroblue tetrazolium (NBT) by using the xanthine/xanthine oxidase system as a superoxide generator. One unit of SOD was defined as the amount of enzyme that caused 50% inhibition of the NBT reduction rate. Activity was expressed as units per milligram protein. The contents of MDA and SOD in the testicular tissue of each group were determined according to the manufacturer's protocol.

### 2.7. Determination of Sperm Counts

The right epididymis of the experimental rat was cut into fragments in a balanced salt solution, and the supernatant was obtained 5 min later. Density gradient centrifugation was performed. The supernatant was obtained, diluted with 2 ml of sperm washing medium (SAGE, UK), and centrifuged at 2,000 rpm for 10 min, and the pellet was diluted with 0.3 ml of fertilization medium (SAGE, UK). Sperm counting was performed under a light microscope using a drop of dilution which was diluted with the fertilization medium described above.

### 2.8. Western Blot Analysis

The testicular tissue samples were homogenized, and protein quantifications were performed using a BCA protein assay kit (Beyotime, Shanghai, China). Equal amounts of protein per specimen were subjected to 10% sodium dodecyl sulfate-polyacrylamide gel electrophoresis and transferred onto polyvinylidene difluoride membranes (0.2 *μ*l or 0.45 *μ*m) (Millipore Corporation, Bedford, MA, USA). The membranes were blocked with 5% fat-free milk for 2 h at room temperature, followed by incubation with the following antibodies at 4°C overnight: nuclear factor like-2 factor (Nrf2; Abcam 1 : 1,000), HO-1 (Abcam 1 : 1,000), quinone oxidoreductase-1 (NQO-1; Abcam 1 : 1,000), NF-*κ*B (CST 1 : 1,000), necrosis factor alpha (TNF-*α*) (CST 1 : 1,000), interleukin-1 beta (IL-1*β*) (CST 1 : 1,000), interleukin-6 (IL-6) (CST 1 : 1,000), and beta-actin (Bioworld 1 : 10,000). After washing with Tris-buffered saline and Tween 20 (TBS-T), the blots were incubated with the horseradish peroxidase-conjugated goat anti-rabbit IgG antibody (BioSharp, Technology Inc., China) at room temperature for 2 h. Finally, protein bands were detected using Image Lab Software and analyzed using Image-Pro Plus software. All the experiments were repeated at least 3 times.

### 2.9. Statistical Analysis

All statistical data were presented as the mean ± standard deviation (SD) and analyzed by one-way ANOVA. SPSS 20.0 software was used for statistical analysis and further evaluated with Tukey post hoc analysis for data homogeneous variance. Dunnett's T3 test was performed for post hoc analysis for data heterogeneous variance. *P* values < 0.05 were considered statistically significant. All data were obtained from at least 6 different rats in each group. All data were obtained from independent experiments over three times.

## 3. Results

### 3.1. Evaluation of Islet Transplantation and Model Building

**Figure 1:**
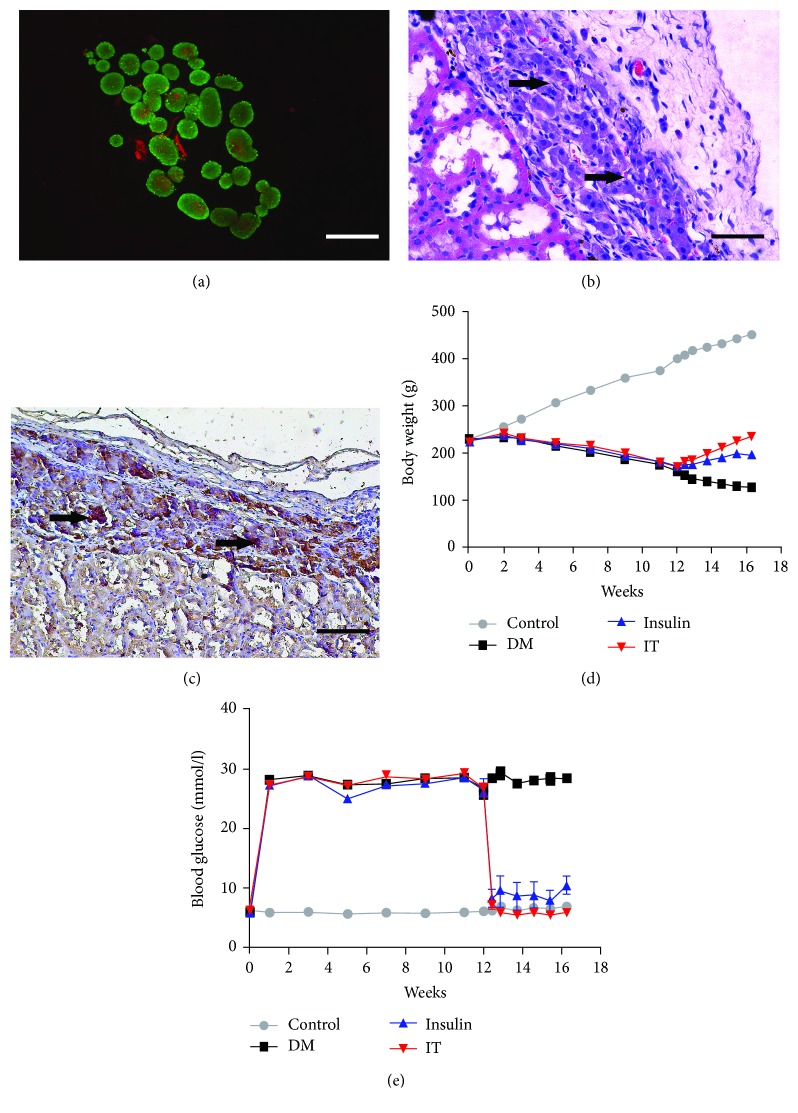
Evaluation of isolated islet viability and function and change in blood glucose in each group. (a) Viability evaluation of isolated islets (FDA-PI staining: ×200). Bar = 50 *μ*m. (b) Stable colonization of transplanted islets under the kidney capsule (HE staining: ×200). Bar = 50 *μ*m. (c) Massive release of insulin after transplantation (immunohistochemical staining: ×200). Bar = 50 *μ*m. (d) Body weight changes over 16 weeks for each group. (e) Rat blood glucose changes over 16 weeks for each group (islet transplantation model rats established at 12 weeks). DM: diabetes mellitus; INS: insulin treatment; IT: islet transplantation.

Highly purified pancreatic islet cells were isolated from donor rat pancreas. FDA-PI staining confirmed high viability in an aliquot of islets before transplantation (>99%, Figure 1(a)). HE staining demonstrated uniform distribution of islets under the renal capsule, and immunohistochemical staining revealed normal insulin secretion after 4 weeks of IT (Figures 1(b) and 1(c)). The body weight of rats in the DN group decreased continuously, while the body weight of rats in the IT group and the INS group increased gradually after treatment. The growth curve of the IT group was significantly higher than that of the INS group (Figure 1(d)). Blood glucose monitoring showed that the diabetic rats sustained hyperglycemia, while diabetic rats treated with insulin or IT showed a significant decrease in the blood glucose level. However, compared with the IT group, the INS group showed a considerable fluctuation in the blood glucose level. The blood glucose level of the IT group was consistently stable in the normal state, suggesting that IT performs better in lowering and stabilizing the blood glucose level compared with the INS group. (Figure 1(e)).

### 3.2. Islet Transplantation Attenuated Testicular Histopathology Damage and Increased Sperm Counts in Diabetes-Induced Rats

**Figure 2:**
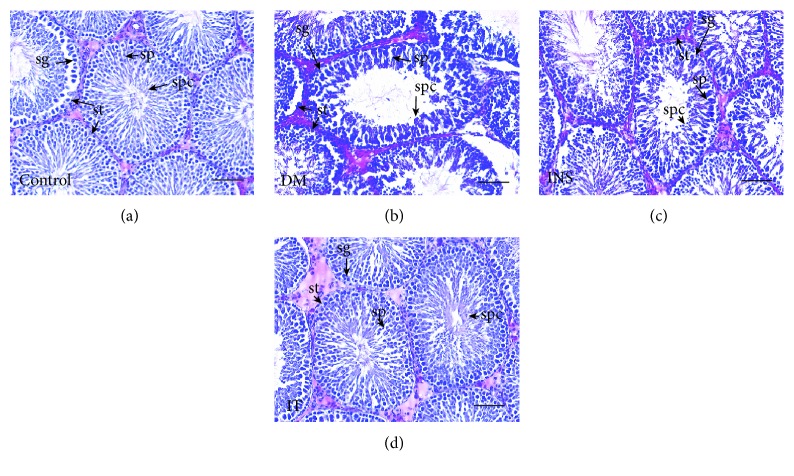
Islet transplantation treatment improved pathological lesions in diabetic rat testes. Representative images of testicular histological examination in each group (*n* = 6 for each group) (HE staining: ×200). Bar = 50 *μ*m. (a) Testicular slices of the control group showed normal spermatogonia (sg), spermatocytes (sp), seminiferous tubules (st), and sperm cells (spc). (b) Testicular slices of the DM group showed spermatogonial cells, spermatocytes, and sperm cells that were degenerated and vacuolated, as well as severely reduced germ cells. (c, d) Testicular slices of the IT group demonstrated more significant recovery of testicular structures compared with that of the INS group. DM: diabetes mellitus; INS: insulin treatment; IT: islet transplantation.

**Figure 3:**
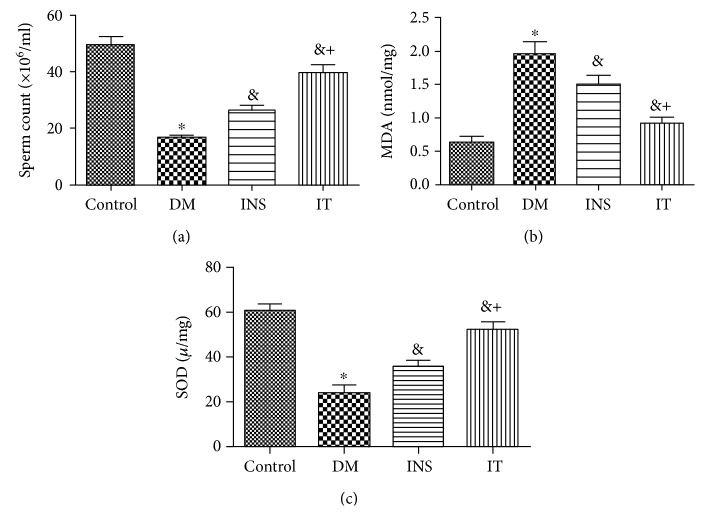
Islet transplantation treatment alleviated sperm count, MDA level, and SOD activity in the testicular tissue of diabetic rats. (a) The number of sperm in the DM group significantly decreased compared with that in the control group. The IT group showed more significant recovery of sperm cells compared with that in the INS group (*n* = 6 for each group). (b) MDA contents in the DM group increased more extensively than that in the control group. Compared with that of the INS group, the MDA concentration of the IT group decreased more significantly (*n* = 6 for each group). (c)The SOD activity in the DM group was significantly lower than that in other groups, and the IT group maintained significantly higher levels of SOD activity compared with that of the INS group (*n* = 6 for each group). DM: diabetes mellitus; INS: insulin treatment; IT: islet transplantation. ^∗^*P* < 0.05 vs. control; ^&^*P* < 0.05 vs. DM; ^+^*P* < 0.05 vs. INS.

Histochemical staining with HE showed that spermatogonial cells, spermatocytes, and sperm cells are degenerated and vacuolated, seminiferous tubules are smaller, and sperm cells are fewer in the DM group (Figures 2(a) and 2(b)) in comparison with those of the other groups. The IT group achieved significant repair of abnormal structures in the testes compared with the DM group, whereas abnormal structures in the INS group were only slightly normalized (Figures 2(b)–2(d)). The sperm count was significantly lower in the DM group compared to the control group. Insulin treatment significantly increased sperm count. The IT group, however, demonstrated more extensive recovery of sperm count than the INS group (Figure 3(a)).

### 3.3. Islet Transplantation Improved Oxidative Stress Levels in the Testes

MDA levels in the DM group increased compared with those in the other groups. Interestingly, the MDA concentration in the IT group decreased significantly in contrast to INS treatment (Figure 3(b)). The activity of SOD in the DM group was also noted to be much lower than the control group; IT and INS treatment significantly improved this change, while the IT group demonstrated significantly higher levels of SOD activity than the INS group (Figure 3(c)).

### 3.4. Islet Transplantation Activated the Nrf-2/HO-1 Pathway in Diabetes-Induced Rats

**Figure 4:**
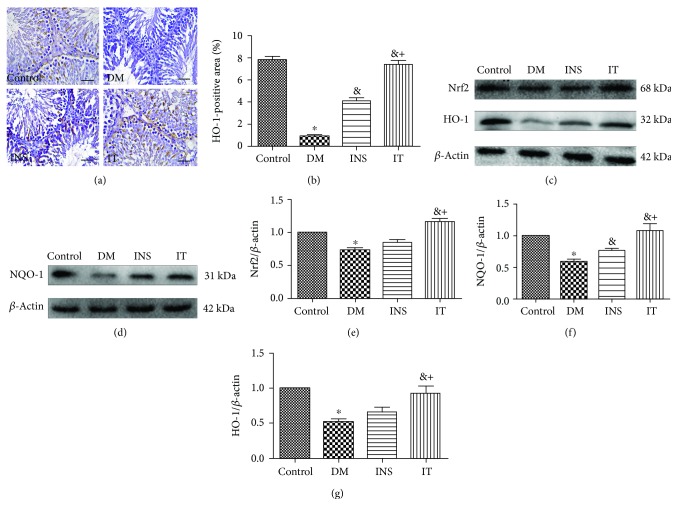
Islet transplantation treatment activated the Nrf-2/HO-1 pathway in the testicular tissues of diabetes-induced rats. (a, b) Immunohistochemical staining (×400) (bar = 25 *μ*m) and quantitative analysis of positive regions showing the expressions and typical distribution of HO-1 in testicular sections. Compared with those of the INS and DM groups, the expression level of HO-1 significantly increased after IT (*n* = 6 for each group). (c–g) Representative Western blot images of Nrf2, HO-1, and NQO1 and quantifications of their expression levels in testicular tissues. DM induced a marked decrease in these parameters. Treatment by IT significantly increased the expression of Nrf2, HO-1, and NQO1. The expressions of these proteins in the INS group were only slightly altered (*n* = 6 for each group). DM: diabetes mellitus; INS: insulin treatment; IT: islet transplantation. ^∗^*P* < 0.05 vs. control; ^&^*P* < 0.05 vs. DM; ^+^*P* < 0.05 vs. INS.

We examined the expression of Nrf2/HO-1 in each group (Figures 4(a)–4(g)). It is known that hyperglycemia results in significant reduction of the Nrf2 expression and its downstream proteins (HO-1, NQO-1). In contrast to the INS and DM groups, the expression of HO-1 in the IT group significantly increased, as determined by immunohistochemical staining (Figures 4(a) and 4(b)). We examined the expression of Nrf2, HO-1, and NQO-1 in testicular tissues by Western blot analysis (Figures 4(c)–4(g)) and observed that IT treatment significantly increased the levels of these proteins. IT leads to a higher degree of activation of the Nrf2/HO-1 pathway compared to INS treatment.

### 3.5. Islet Transplantation Inhibited Diabetes-Induced Testicular Inflammation

**Figure 5:**
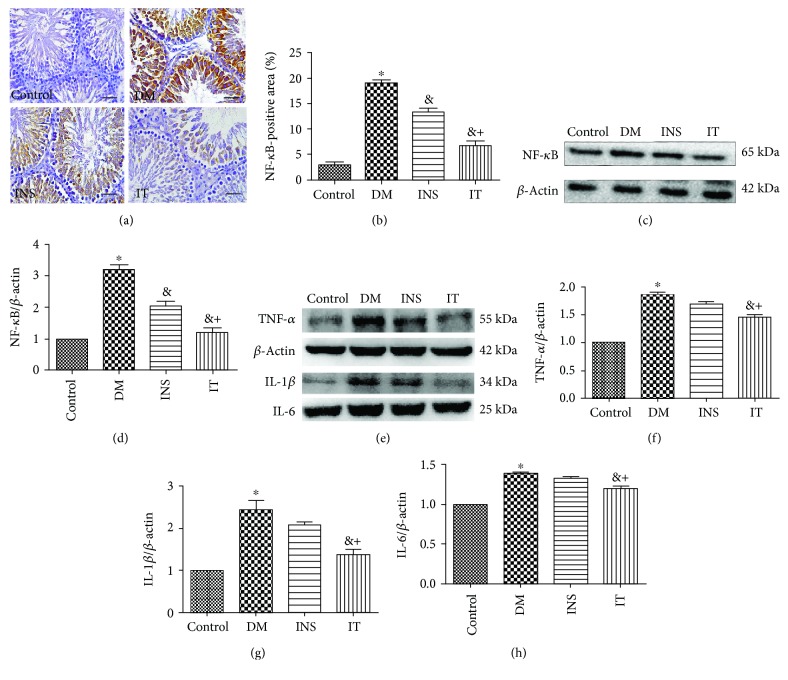
Islet transplantation treatment inhibited NF-*κ*B expression in diabetic rats. (a, b) NF-*κ*B levels measured by immunohistochemical staining (×400) (bar = 25 *μ*m) and quantitative analysis of positive regions showed that the increase in NF-*κ*B was significantly improved in the INS group compared with that in the control group; IT attenuated the expression of the protein (*n* = 6 for each group). (c, d) Western blot images of NF-*κ*B and quantitative analysis of its expression levels in the DM group revealed significant upregulation. By contrast, NF-*κ*B in the IT group was significantly downregulated compared with that in the INS group (*n* = 6 for each group). (e–h) Typical Western blot images of TNF-*α*, IL-1*β*, and IL-6 intesticular tissues and quantitative analysis of their expression levels. DM caused a significant increase in the above parameters. The IT treatment group significantly reduced the expression of TNF-*α*, IL-1*β*, and IL-6. The expression of the above proteins in the INS group was only slightly altered (*n* = 6 for each group). DM: diabetes mellitus; INS: insulin treatment; IT: islet transplantation. ^∗^*P* < 0.05 vs. control; ^&^*P* < 0.05 vs. DM; ^+^*P* < 0.05 vs. INS.

To explore the effect of IT on inflammation, we measured the protein expression level of NF-*κ*B by immunohistochemical staining and Western blot analysis (Figures 5(a)–5(d)), and quantitatively analyzed the protein expression levels of TNF-*α*, IL-1*β*, and IL-6 (Figures 5(e)–5(h)). The DM group showed a significant increase in NF-*κ*B, TNF-*α*, IL-1*β*, and IL-6 levels, whereas the IT and INS groups attenuated the expression of these proteins. Intriguingly, treatment with the IT group suppressed these parameters more significantly than the INS group. Concordant results were obtained by immunohistochemical staining and Western blot analysis of NF-*κ*B.

## 4. Discussion

Research findings report that persistent hyperglycemia can cause testicular tissue damage [22]. The seminiferous tubules may become smaller, in addition to degeneration and vacuolation of the spermatogonia, spermatocytes, and sperm cells [23]. Previous studies have also indicated that DM decreases the number of Sertoli and Leydig cells, disrupts the germinal epithelium, and disturbs the process of spermatogenesis, ultimately reducing the production of sperm cells and affecting fertility [24, 25]. As expected, our results showed that hyperglycemia reduces sperm cell counts and alters the histopathology of the testes. We demonstrated that IT treatment could normalize blood glucose levels and better restore sperm production, testicular structures, and functions compared with INS therapy. Furthermore, we observed that the protective properties of IT appear to be associated with suppression of oxidative stress and inflammation via activating the Nrf-2/HO-1 pathway and inhibiting activation of NF-*κ*B.

IT represents a promising curative treatment for patients with type 1 diabetes. Compared with endocrine replacement therapies, such as exogenous INS treatment, the main advantage of IT is that it can maintain insulin secretory capacities and improve insulin sensitivity and glucose tolerance by restoring the function of *β* cells [26–29]. Insulin release/sensitivity is extremely important for testes because this process controls the metabolic cooperation and metabolism-associated gene expression between testicular cells [30, 31]. IT counteracts hyperglycemia, hypoinsulinemia, and insensitivity that is characteristic of diabetic individuals. These are critical to the normal occurrence of spermatogenesis [32, 33].

Evolving evidence suggests that the damage to the male reproductive system caused by hyperglycemia could be explained by a variety of mechanisms [34]. Oxidative stress plays an important role in the development of diabetic complications [35, 36]. Several harmful effects could be observed on the male reproductive system under oxidative stress, including testicular atrophy, decreased weight of the sexual organs, testicular impairments, and even reductions in sperm count. In this research, IT alleviated these injuries possibly on account of its potent antioxidant properties. IT significantly upregulated Nrf2 and increased expressions of HO-1 and NQO-1 and antioxidants, such as SOD, all of which play an important role in antioxidative defense. Nrf2, as a redox-sensitive transcription factor of the antioxidative defense pathway, could induce many cytoprotective factors, such as HO-1 and NQO-1, therefore restoring the redox balance in cells [37–39]. HO-1 exhibits effective protection by activating the oxidative degradation function, which converts highly deleterious free heme into biliverdin, then bilirubin [40]. In vivo, the upregulation of HO-1 also causes reductions in free radical levels and NADPH oxidase activity [41]. NQO-1 regulates the cellular stress response by reducing quinone [42]. Increased antioxidant enzymes, such as SOD, can scavenge free radicals or reactive oxygen intermediates into nonradical products in male reproductive organs [43]. In a previous study, Negri et al. showed that SOD-based antioxidant supplementation exerts a beneficial effect on reducing sperm DNA fragmentation and maintaining sperm DNA integrity [44]. These measures protect various cells, such as spermatogonial cells, spermatocytes, and sperm cells, against free radical damage. The damage induced by free radicals to cells can be determined by measuring levels of MDA, the ultimate product of unsaturated LPO [43]. As expected, the MDA level of the IT group was nearly normal, thereby indicating marked reduction of free radical-induced membrane injury; in contrast, MDA levels in the INS group were only slightly restored. Although INS play a favorable role in blood glucose control, its ability to activate the antioxidant pathway in the testes is much lower than that of IT. This may be one of the main reasons for delayed recovery of testicular injury with INS treatment. Therefore, one of the favorable effects of IT in retarding diabetes-induced testicular dysfunction and impairment of spermatogenesis might be attributed to its ability to regulate blood glucose and its strong antioxidant level.

The role of inflammation in testicular injury should not be ignored. The results of the findings indicate that diabetes mediates testicular damage by increasing the expression of NF-*κ*B which elicit inflammatory cytokine activities, such as those of IL-6, TNF-*α*, and IL-1*β*, to promote inflammatory responses [45]. In our study, we observed that IT could significantly inhibit the NF-*κ*B-mediated inflammation of spermatogonia and spermatocytes. Similar to previous results, the expression of NF-*κ*B in the INS group was significantly higher than that in the control and IT groups. The above results indicate that the protective properties of IT appear to be accomplished in part by downregulation of NF-*κ*B.

Several reports have shown that free radicals can significantly influence the NF-*κ*B pathway, therefore inducing inflammatory cytokine activation [46–48]. Interestingly, expression of cytokines is able to generate severe oxidative stress [49]. In fact, macrophages, Sertoli cells, and Leydig cells produce nitric oxide (NO) primarily under hypoxic and inflammatory conditions, after which high levels of NO interact with superoxide anions, resulting in the formation of peroxynitrite and peroxynitrous acid [50]. These processes may promote cell damage, which could lead to inflammation and/or production of other free radicals [51]. The crosslinks between inflammation, oxidative stress, sperm cell production, and testicular injury should be investigated in future work.

## 5. Conclusion

In conclusion, our study confirmed that islet transplantation exerts protective effects on testicular injury in the STZ-induced rat model of type 1 diabetes. IT may protect against damage to the testicular tissue microstructure and interference of spermatogenesis by inhibiting oxidative stress and inflammation through adjusting the Nrf-2/HO-1 and NF-*κ*B pathways. These findings provide a potential method for addressing testicular injury and infertility in diabetes patients. However, the effects of other factors such as the environment of sperm development and other endocrine factors on testicular injury and spermatogenesis remain unclear; further research on these topics is necessary.

## Data Availability

The data used to support the findings of this study are available from the corresponding authors upon request.

## Abbreviations

DM: Diabetes mellitus
STZ: Streptozotocin
LPO: Lipid peroxidation
FDA-PI: Fluorescein diacetate-propidium iodide
HE: Hematoxylin-eosin
DAB: Diaminobenzidine
MDA: Malondialdehyde
SOD: Superoxide dismutase
Nrf-2: Nuclear factor like-2 factor
HO-1: Heme oxygenase-1
NQO-1: Quinone oxidoreductase-1
NF-*κ*B: Nuclear factor kappa B.
